# Differential Effects of Prostaglandin D_2_ Signaling on Macrophages and Microglia in Murine Coronavirus Encephalomyelitis

**DOI:** 10.1128/mBio.01969-21

**Published:** 2021-09-07

**Authors:** Abhishek Kumar Verma, Jian Zheng, Matthias Mack, Florent Ginhoux, Stanley Perlman

**Affiliations:** a Department of Microbiology and Immunology, University of Iowa, Iowa City, Iowa, USA; b Department of Internal Medicine II, University Hospital Regensburg, Regensburg, Germany; c Shanghai Institute of Immunology, Department of Immunology and Microbiology, Shanghai Jiao Tong University School of Medicine, Shanghai, China; d Singapore Immunology Network, Agency for Science, Technology and Research, Singapore, Singapore; e Translational Immunology Institute, SingHealth Duke-NUS Academic Medical Centre, Singapore, Singapore; Johns Hopkins Bloomberg School of Public Health

**Keywords:** macrophage, encephalitis, phagocytosis, PGD_2_/DP1 axis, microglia, Ms4a3-cre, mouse hepatitis virus (MHV), coronavirus

## Abstract

Microglia and macrophages initiate and orchestrate the innate immune response to central nervous system (CNS) virus infections. Microglia initiate neurotropic coronavirus clearance from the CNS, but the role of infiltrating macrophages is not well understood. Here, using mice lacking cell-specific expression of DP1, the receptor for prostaglandin D_2_ (PGD_2_), we delineate the relative roles of PGD_2_ signaling in microglia and macrophages in murine coronavirus-infected mice. We show that the absence of PGD_2_/DP1 signaling on microglia recapitulated the suboptimal immune response observed in global DP1^−/−^ mice. Unexpectedly, the absence of the DP1 receptor on macrophages had an opposite effect, resulting in enhanced activation and more rapid virus clearance. However, microglia are still required for disease resolution, even when macrophages are highly activated, in part because they are required for macrophage recruitment to sites of infection. Together, these results identify key differences in the effects of PGD_2_/DP1 signaling on microglia and macrophages and illustrate the complex relationship between the two types of myeloid cells.

## INTRODUCTION

Given the inability of neurons to regenerate, it is critical that invading pathogens be eliminated quickly and efficiently to minimize central nervous system (CNS) damage. Tissue destruction is mediated by both virus replication and the host antivirus immune response (1, 2), emphasizing the importance of a finely tuned immune response with finely calibrated proinflammatory and anti-inflammatory components. A number of studies have documented the role of CNS-resident myeloid cells (microglia and macrophages) in directing these antiviral responses (3–5). For instance, in the context of infection with neurotropic viruses such as mouse hepatitis virus (MHV), West Nile virus, dengue virus, herpes simplex virus, or Theiler’s encephalomyelitis virus, the depletion of microglia prior to or at the time of infection results in worse outcomes (6–8).

We and others have shown that eicosanoids with anti-inflammatory/proresolving effects are necessary to minimize tissue damage during viral infections of the CNS (4, 9–11). Specifically, prostaglandin D_2_ (PGD_2_), the most abundant prostaglandin in murine brain (12), generally mediates an anti-inflammatory effect when signaling through the DP1 receptor on myeloid cells (13–15). We have previously shown that the global ablation of the DP1 receptor caused a dysregulated immune response after infection with the neurotropic JHM strain of MHV (JHMV), characterized by a delayed type I interferon (IFN-I) response concomitant with an exaggerated interleukin-1β (IL-1β) response. This dysregulated immune response appeared to largely result from microglia-specific effects and resulted in higher viral loads and delayed virus clearance (15). Of note, PGD_2_/DP1 signaling is most often anti-inflammatory in other tissues such as the lungs of aged mice infected with pathogenic human coronaviruses, where it may inhibit the development of an effective antiviral immune response (16). Together, these results indicate that the consequences of PGD_2_/DP1 signaling are multifaceted and context dependent, and one cannot readily categorize it as either anti- or proinflammatory.

Contrasting roles have been attributed to microglia and macrophages in several diseases of the CNS. In one study of mice with experimental autoimmune encephalitis (EAE), macrophages were implicated in the initiation of demyelination, while microglia affected myelin debris removal (17). Macrophages have been shown to reduce microglial phagocytosis and activation in the setting of spinal cord injury (4). Macrophages have also been reported to promote inflammation and neuronal injury in seizure models of Theiler’s murine encephalomyelitis virus (TMEV) (18). Given all of these disparate roles for microglia and macrophages in various pathological settings and the protective role of PGD_2_/DP1 signaling in the CNS, we sought to assess the effects of DP1 ablation specifically on these two cell types. To this end, we obtained mice floxed for the DP1 receptor (PTGDR^flox^; C57BL/6J background) and crossed them with mice expressing B6·129P2-Cx3cr1^tm2.1(cre/ERT2)Litt^/WganJ-cre (CX3CR1-DP1^−/−^) or B6.129P2-*Lyz2^tm1^*^(^*^cre^*^)^*^lfo^*/J (Lyz2-DP1^−/−^). CX3CR1-cre is expressed only after tamoxifen treatment and largely on microglia. Lysozyme M (LysM), the protein product of *Lyz2*, is primarily found on macrophages and neutrophils (19–21). We infected these mice with a recombinant version of JHMV (rJHMV) and found that infection of CX3CR1-DP1^−/−^ mice (microglia-specific DP1 ablation) resulted in a dysregulated immune response and increased mortality, effectively phenocopying rJHMV-infected mice with global depletion of DP1 expression. However, in marked contrast, infection of Lyz2-DP1^−/−^ (macrophage/neutrophil-specific DP1 ablation) mice resulted in more rapid viral clearance and less clinical disease. Of note, we also show that the primary effect of DP1 signaling in these mice is on macrophages and not neutrophils. Together, these results demonstrate the very different roles that PGD_2_/DP1 signaling has in two closely related myeloid cell populations in the CNS.

## RESULTS

### Different outcomes in rJHMV-infected mice after macrophage- and microglia-specific DP1 ablation.

**FIG 1:**
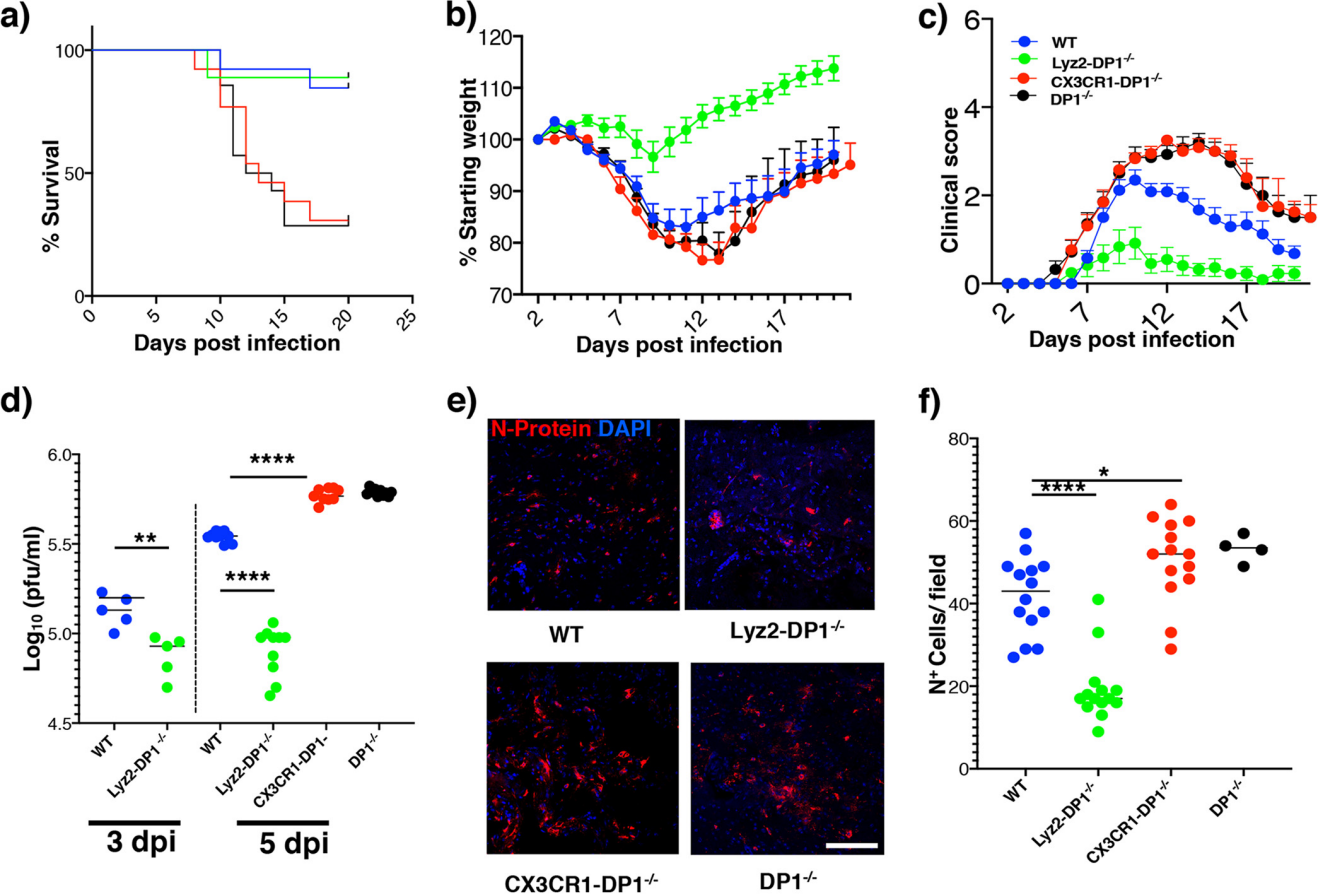
PGD_2_/DP1 signaling has contrasting roles in different cell types. (a to c) Disease progression in WT, Lyz2-DP1^−/−^, CX3CR1-DP1^−/−^, and DP1^−/−^ mice infected with 700 PFU of rJHMV intracranially. Data are pooled from three independent experiments (*n *=* *12 to 14 mice per group). A log rank Mantel-Cox test was used for survival analysis between Lyz2-DP1^−/−^, CX3CR1-DP1^−/−^, and DP1^−/−^ mice (**, *P* < 0.01). Mann-Whitney U tests were used to analyze weight (days 8 to 20) (*P* < 0.05) (b) and clinical score (days 8 to 18) (*P* < 0.05) (c) data between Lyz2-DP1^−/−^, CX3CR1-DP1^−/−^, and DP1^−/−^ mice. (d) Virus titers in the brains of mice at 3 dpi and 5 dpi show reduced virus loads in Lyz2-DP1^−/−^ compared to WT mice. Data represent the means ± SEM of results pooled from two independent experiments with 5 to 10 mice per group. Data were analyzed using Mann-Whitney U tests. ****, *P* < 0.0001. (e) Representative confocal images showing virus distribution in the brain, as assessed by staining for N protein. (f) Summary data, analyzed using Mann-Whitney U tests. Five sections from 3 individual mice were included in the analysis. Data are representative of results from 3 mice per group (means ± SEM). Bar, 50 μm.

We developed mice lacking cell type-specific expression of the PGD_2_/DP1 receptor as described above. To restrict DP1 ablation to microglia, we crossed CX3CR1-cre mice with floxed DP1 mice, recognizing that CX3CR1 is also expressed on a subset of peripheral macrophages and, to a lesser extent, on neurons, and treated them with tamoxifen. To avoid the effects of CX3CR1-mediated expression in peripheral macrophages, mice are often rested for 6 to 8 weeks after tamoxifen treatment to allow the replacement of DP1-deleted macrophages by wild-type (WT) cells (22, 23). However, this is not possible in studies of neuroattenuated rJHMV because infection is most consistent when mice are infected at 6 weeks of age (24, 25). WT mice infected with rJHMV developed mild encephalitis characterized by moderate weight loss and clinical scores (Fig. 1a to c). In contrast, as reported previously (15), DP1^−/−^ mice developed severe disease characterized by approximately 70% mortality. Infection of CX3CR1-DP1^−/−^ mice with rJHMV resulted in approximately 60 to 70% mortality, mirroring global DP1 absence. Increased mortality in infected CX3CR1-DP1^−/−^ mice was associated with enhanced weight loss (Fig. 1b) and higher clinical scores (Fig. 1c). In contrast, Lyz2-DP1-specific deletion enhanced resistance to rJHMV infection, with reduced mortality and significantly less weight loss and morbidity than in infected DP1^−/−^ mice (Fig. 1a to c), highlighting the contrasting effects of DP1 signaling on the 2 different types of myeloid cells.

In agreement with the lack of clinical signs, Lyz2-DP1^−/−^ mice showed at least 10-fold-lower virus titers than CX3CR1-DP1^−/−^, DP1^−/−^, or WT mice, with CX3CR1-DP1^−/−^ and DP1^−/−^ mice harboring the highest virus loads (Fig. 1d) at 5 days postinfection (dpi), the peak of virus replication. Virus titers were also lower at 3 dpi in Lyz2-DP1^−/−^ than in WT mice, demonstrating the early control of infection in mice lacking macrophage-specific DP1 expression. To further examine the distribution of virus in the brain, we performed confocal microscopy. While we observed no differences in virus localizations within the brain, Lyz2-DP1^−/−^ mice exhibited significantly fewer infected cells at each site of infection, as assessed by nucleocapsid (N) protein staining (Fig. 1e and f). In contrast, in agreement with the observed increase in viral loads, brains from CX3CR1-DP1^−/−^ and DP1^−/−^ mice showed increased staining for virus antigen (Fig. 1e and f). These data indicate that while PGD_2_/DP1 signaling on microglia is protective, its signaling on macrophages/neutrophils appears to have a deleterious effect.

### DP1 signaling modulates cytokine and chemokine expression by myeloid cells.

**FIG 2:**
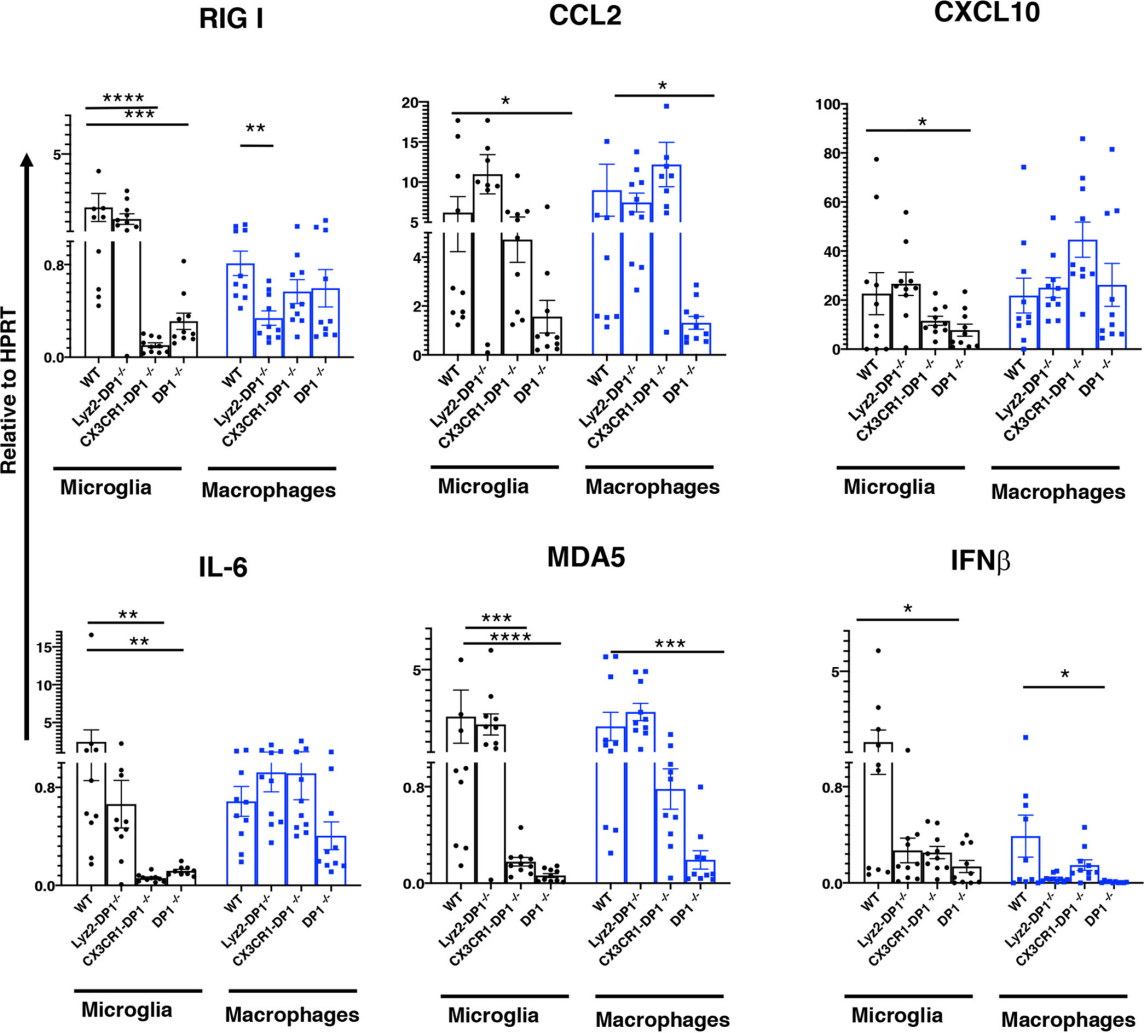
Cytokine and chemokine expression in infected mice. Cytokine and chemokine mRNA expression was examined in sorted microglia (black) and macrophages (blue) from rJHMV-infected brains of WT, Lyz-DP1^−/−^, CX3CR1-DP1^−/−^, and DP1^−/−^ mice at 3 dpi. Gene expression was normalized to HPRT. Pooled data from three independent experiments with 10 to 12 mice per group were analyzed by a Mann-Whitney U test (means ± SEM). *, *P* < 0.05; **, *P* < 0.01; ***, *P* < 0.001; ****, *P* < 0.0001.

Given the well-described anti-inflammatory role of DP1 signaling and the increases in virus loads in the global or microglia-specific absence of DP1, we reasoned that the PGD_2_/DP1 signaling axis diminished the antivirus immune response (Fig. 2). In JHMV-infected DP1^−/−^ mice, contrary to this expectation, we observed diminished expression of molecules such as RIG-I, CCL2, CXCL10, IL-6, MDA5, and IFN-β, with effects being most prominent in microglia. Similarly, infection of CX3CR1-DP1^−/−^ mice resulted in diminished expression of RIG-I, IL-6, MDA5, and CXCL10 in microglia, compared to WT mice, although CXCL10 did not reach statistical significance (*P* = 0.09). DP1 signaling in CX3CR1-DP1^−/−^ macrophages was normal, and consistent with this, there were no differences in the macrophage-specific expression of these proinflammatory molecules. On the other hand, the absence of DP1 signaling in macrophages had little effect on the expression of these molecules in either macrophages or microglia, with the exception of a reduction in IFN-β expression in macrophages. Together, these results suggest that DP1 signaling in microglia is critical for normal cytokine and chemokine expression by these cells, likely contributing to poor outcomes. However, the effects of these molecules in Lyz2-DP1^−/−^ mice on expression were limited, suggesting that they did not make major contributions to the improved outcomes observed after rJHMV infection.

### Macrophages lacking DP1 expression are highly activated and exhibit enhanced phagocytic activity.

**FIG 3:**
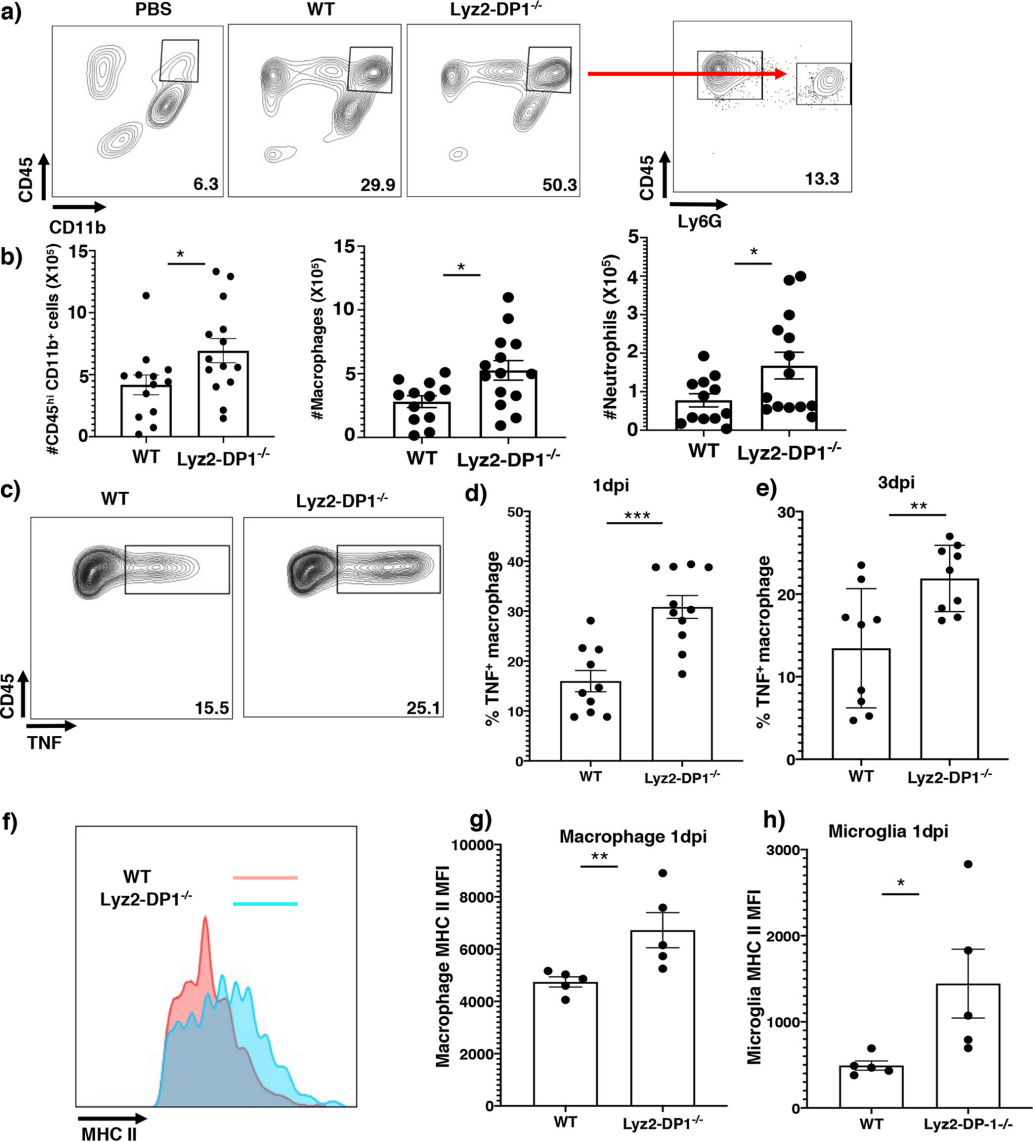
Enhanced recruitment with increased TNF and MHC-II expression by Lyz2-DP1^−/−^ macrophages. (a) WT and Lyz2-DP1^−/−^ mice were infected with 700 PFU rJHMV or treated with PBS, and brains were harvested at 24 h p.i. The frequencies of macrophages are shown. (b) Numbers of macrophages and neutrophils in the brain. Pooled data from three independent experiments with 10 to 12 mice per group and analysis by Mann-Whitney U tests are shown (means ± SEM). Macrophages and microglia from Lyz2-DP1^−/−^ and WT mice were harvested at 1 and 3 dpi and analyzed for the expression of MHC-II and TNF directly *ex vivo* in the absence of additional stimulation. (c to e) Flow cytometric analysis of cells isolated from infected brains at 1 dpi and 3 dpi showing increased expression of TNF in macrophages. (d and e) Summary data from two pooled independent experiments with 9 to 10 mice per group. Data were analyzed using Mann-Whitney U tests (means ± SEM). **, *P* < 0.01; ***, *P* < 0.001. (f) Representative flow cytometric plots at day 1 p.i. (g and h) Bar graphs showing mean fluorescence intensities at day 1 p.i. for macrophages (g) and microglia (h). Each data point in bar graphs represents an individual mouse. Data are from a single experiment and are representative of results from two individual experiments. The data represent the means ± SEM and were analyzed using Mann-Whitney U tests. *, *P* < 0.05; **, *P* < 0.01; ***, *P* < 0.001.

The expression of proinflammatory molecules (Fig. 2) did not explain why Lyz2-DP1^−/−^ mice were better protected than the other strains. To interrogate whether macrophages in Lyz2-DP1^−/−^ mice were functionally different from cells isolated from the other strains, we assessed their activation status and phagocytic properties. By as early as 1 dpi, the total numbers of CNS-infiltrating cells, including macrophages (CD45^+^ CD11b^+^), were significantly higher in Lyz2-DP1^−/−^ than in WT infected mice (Fig. 3a and b). These differences were resolved by 3 dpi. Compared to those from WT mice, macrophages but not microglia (see Fig. S1a and b in the supplemental material) from Lyz2-DP1^−/−^ mice showed enhanced activation as evidenced by the higher capacity to secrete tumor necrosis factor (TNF) at both 1 and 3 dpi (Fig. 3c to e). Notably, such differences were not observed when WT, CX3CR1-DP1^−/−^, and DP1^−/−^ mice were compared at 1 and 3 dpi (Fig. S1c to g). Additionally, both microglia and macrophages from Lyz2-DP1^−/−^ mice expressed significantly higher levels of major histocompatibility complex class II (MHC-II) (Fig. 3f to h). Notably, the numbers of virus-specific CD4 (and CD8) T cells in the brain did not differ when WT, Lyz-DP1^−/−^, and CX3CR1-DP1^−/−^ mice were compared (Fig. S2). Collectively, these data show that macrophages exhibited higher activation and antigen presentation potential when they were devoid of DP1 signaling but that this did not affect T cell numbers.

10.1128/mBio.01969-21.1 FIG S1 Microglia do not show an activated profile. (a and b) Microglia from Lyz2-DP1^−/−^ mice harvested at 1 and 3 dpi do not exhibit increased TNF expression. Myeloid cell responses in brains of WT, CX3CR1-DP1^−/−^, and DP1^−/−^ mice were assessed. Cells were harvested from the brains of MHV-infected WT, CX3CR1-DP1^−/−^, or DP1^−/−^ mice at 1 dpi. (c to e) Numbers of macrophages and neutrophils in the brain 1 day after rJHMV infection in WT, CX3CR1-DP1^−/−^, and DP1^−/−^ mice. (e to g) Macrophage and neutrophil cell numbers at 3 dpi in WT and Lyz-DP1^−/−^ mice. Download FIG S1, TIF file, 2.5 MB. Copyright © 2021 Verma et al. 2021 Verma et al. https://creativecommons.org/licenses/by/4.0/ This content is distributed under the terms of the Creative Commons Attribution 4.0 International license.

10.1128/mBio.01969-21.2 FIG S2 CD4 and CD8 T cell responses in rJHMV-infected mice. WT, Lyz-DP1^−/−^, and CX3CR1-DP1^−/−^ mice were infected with rJHMV, and brains were harvested at 7 dpi for quantifying T cell responses. (a to c) Brain cells were incubated with M133 peptide in the presence of brefeldin A for 6 h and assayed for IFN-γ expression by flow cytometry. (d to f) Brain cells were incubated with S510 peptide in the presence of brefeldin A for 6 h and assayed for IFN-γ expression by flow cytometry. Download FIG S2, TIF file, 2.4 MB. Copyright © 2021 Verma et al. 2021 Verma et al. https://creativecommons.org/licenses/by/4.0/ This content is distributed under the terms of the Creative Commons Attribution 4.0 International license.

**FIG 4:**
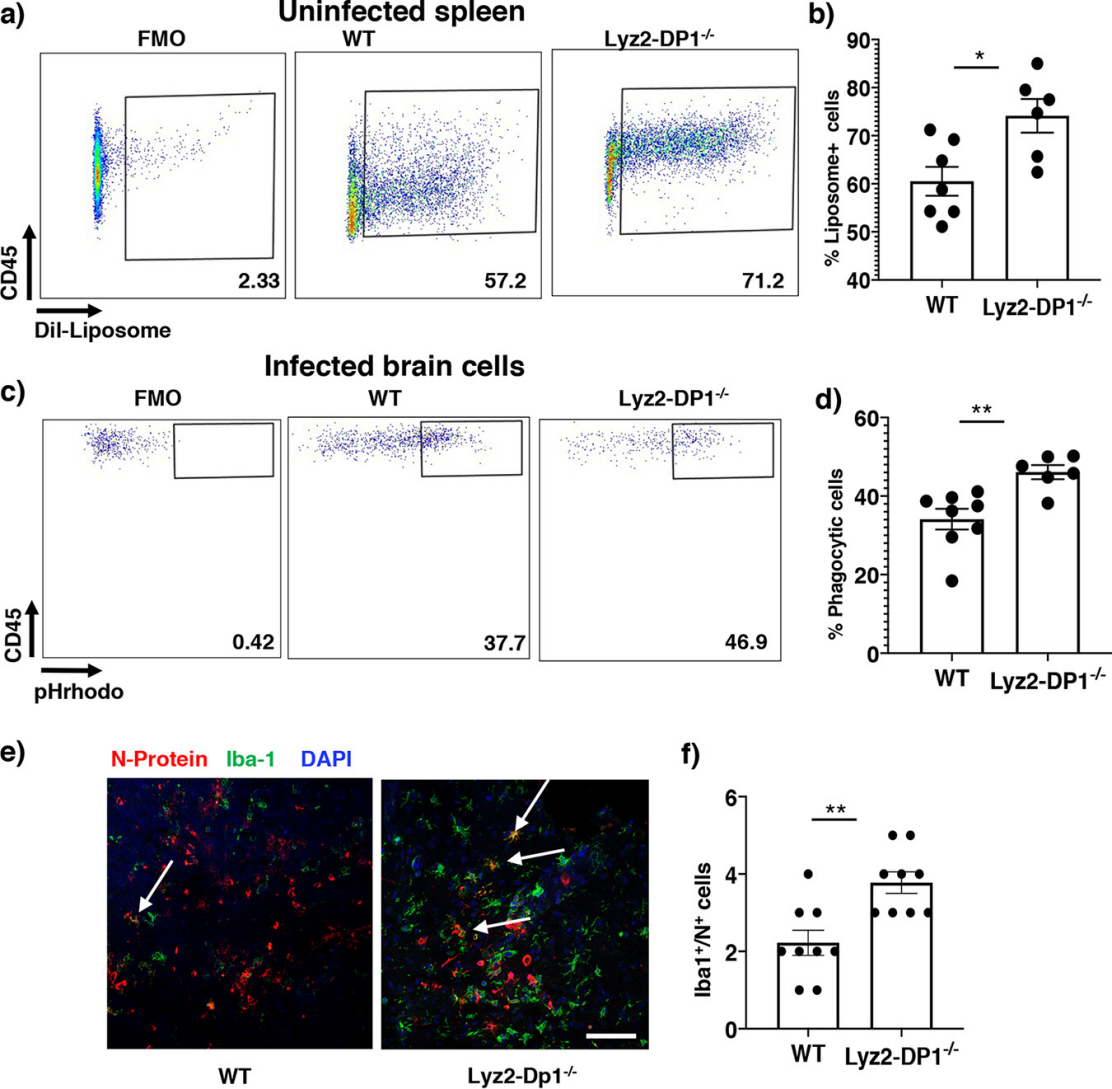
Enhanced phagocytosis in uninfected and infected Lyz2-DP1^−/−^ mice. (a) Splenic phagocytic function was assessed by administering Dil-labeled liposomes or PBS to uninfected mice and assessing uptake in the spleen 20 h after administration. CD45^+^ CD11b^+^ cells were gated and assayed for liposome-dye uptake. (b) Summary data showing the frequency of positive cells. Data are represented as means ± SEM from three independent experiments with 6 to 7 mice per group. (c) Cells were isolated from the CNS of infected WT and Lyz2-DP1^−/−^ mice and exposed to pHrhodo E. coli beads as described in Materials and Methods. FMO (fluorescence minus one) was used to set gates. After 1 h of incubation, CD45^hi^ CD11b^+^ cells were assessed for rhodamine expression by flow cytometry. (d) Representative summary data from two experiments (means ± SEM). (e) Infected brains were harvested and analyzed for Iba1 and N protein expression. The confocal image shows increased numbers of doubly labeled cells, suggestive of enhanced phagocytosis of infected cells. The arrows show double-positive cells. The image is representative of results from an experiment performed with three mice per group. (f) Summary data. Five random fields from each of three mice were analyzed, and the number of double-positive cells was recorded. *, *P* < 0.05; **, *P* < 0.01. Bar, 50 μm.

The enhanced activation and antigen presentation capacity raised the possibility that these macrophages exhibited increased phagocytic function, important for viral clearance (26). Consequently, we next assessed the phagocytic capacity of macrophages by injecting uninfected mice with fluorochrome Dil-labeled liposomes (Dil). After phagocytosis, macrophages will be labeled with Dil, and labeling can be detected by flow cytometry. Twenty hours after liposome injection, spleens were harvested, and cells were analyzed for Dil labeling. Significantly larger numbers of splenic macrophages were Dil labeled in Lyz2-DP1^−/−^ than in WT mice (Fig. 4a and b). To examine whether macrophages from infected mice exhibit an enhanced phagocytic capacity, we isolated cells from the brains of rJHMV-infected Lyz2-DP1^−/−^ and WT mice at 1 dpi and incubated them directly *ex vivo* with Escherichia coli labeled with pH-dependent rhodamine (pHrhodo)-conjugated beads. Fluorescence will be observed only at an acidic pH and would indicate lysosomal uptake in target cells. As in splenic macrophages, CNS-infiltrating macrophages from Lyz2-DP1^−/−^ mice exhibited an enhanced phagocytic capacity compared to those from WT mice, as shown by a higher percentage of macrophages ingesting pHrhodo as well as a higher geometric mean fluorescence intensity (gMFI), indicating the presence of larger numbers of fluorochrome-conjugated E. coli cells on a per-cell basis (Fig. 4c and d). We also used confocal microscopy to analyze the number of Iba-positive (Iba^+^) cells in Lyz2-DP1^−/−^ and WT brains that expressed viral N protein as a measure of virus uptake by these cells. At 3 dpi, we observed that a larger number of Iba^+^ cells were N^+^, consistent with enhanced phagocytosis (Fig. 4e and f). While this result could be consistent with a higher rate of infection of macrophages in Lyz2-DP1^−/−^ mice, we think that this is unlikely since virus loads were lower in Lyz2-DP1^−/−^ mice. Together, these results indicate that macrophages lacking DP1 expression were more activated and phagocytic than those from WT mice.

### Blocking macrophage infiltration into the CNS abrogates the protective effects of DP1 absence.

**FIG 5:**
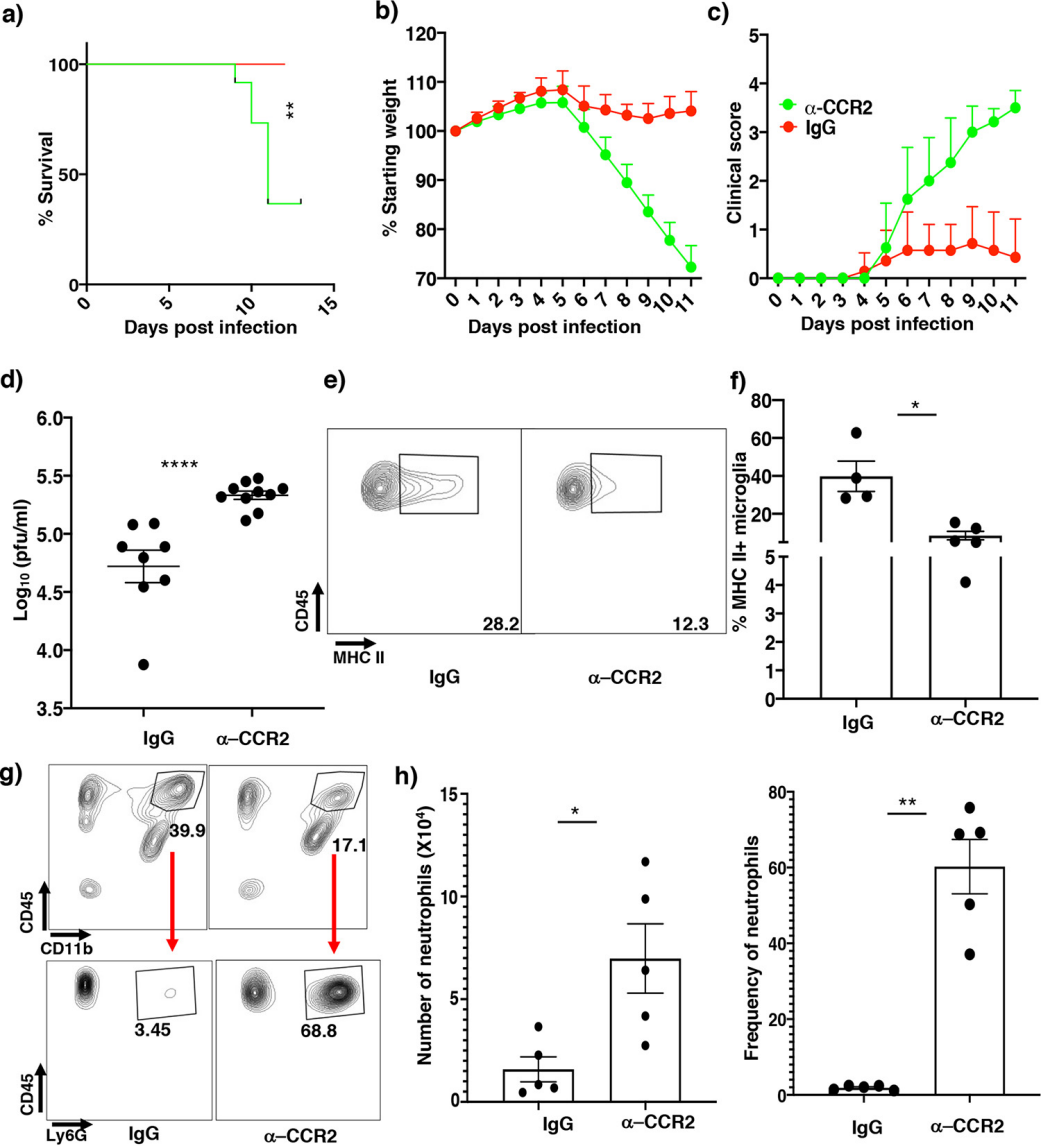
Macrophages play a crucial role in protection against rJHMV-induced encephalitis in Lyz2-DP1^−/−^ mice. (a to c) Infected Lyz2-DP1^−/−^ mice were treated with anti-CCR2 (MC21) or control antibody at −1, 3, and 7 dpi and monitored for survival, weight, and clinical score. MC21 mAb treatment resulted in a marked increase in disease severity in Lyz-DP1^−/−^ mice. A log rank Mantel-Cox test was used to analyze survival (**, *P* < 0.01 [control and anti-CCR2 mAb-treated mice]). Mann-Whitney U tests were used to analyze weight (days 7 to 11) (*P* < 0.05) (b) and clinical score (days 7 to 11) (*P* < 0.05) (c) data. (d) Virus titers in the brain were elevated in MC21- compared to IgG-treated mice. (e) Flow cytometric analysis showing decreased MHC-II frequencies in MC21 mAb-treated Lyz2-DP1^−/−^ mice. (f) Bar graph showing frequencies of MHC-II-positive microglia. (g) Flow cytometry data showing that MC21 antibody treatment affects macrophage but not neutrophil entry into the CNS. (h) Summary data. Data were analyzed by Mann-Whitney U tests. **, *P* < 0.01; ****, *P* < 0.0001.

To verify that macrophages confer enhanced protection in Lyz2-DP1^−/−^ mice, we blocked macrophage infiltration into the infected brain by treating mice with CCR2-blocking (MC21 monoclonal antibody [mAb]) (27, 28) or control antibody 1 day before and 3 and 7 days after infection. Indeed, treatment with MC21 mAb effectively prevented macrophage entry into the brain (Fig. S3a and b). Infected Lyz2-DP1^−/−^ mice receiving MC21 mAb exhibited higher clinical scores, severe weight loss, and nearly 60% mortality, while the control mAb-treated group had minimal signs of clinical disease (Fig. 5a to c). To determine whether the greater morbidity and mortality in MC21 mAb-treated mice were associated with diminished virus clearance, we measured brain virus titers at 5 dpi. Virus titers were higher in mice treated with MC21 than in those treated with the control mAb (Fig. 5d). MC21 mAb treatment also resulted in reduced MHC-II expression by microglia (Fig. 5e and f). Of note, CCR2-blocking antibody did not inhibit the infiltration of neutrophils into the brain, indicating that macrophages were most important for protection in Lyz2-DP1^−/−^ mice (Fig. 5g and h).

10.1128/mBio.01969-21.3 FIG S3 Anti-CCR2 mAb treatment inhibits macrophage infiltration into the CNS after rJHMV infection. Mice were treated with either rat IgG or anti-CCR2 (MC21) mAb. Data show the absence of CD45^hi^ CD11b^+^ Ly6G^−^ cells, demonstrating successful depletion of macrophages. Data represent results for 5 mice per group. A Mann-Whitney U test was used to analyze the data. **, *P* < 0.01. Download FIG S3, TIF file, 2.3 MB. Copyright © 2021 Verma et al. 2021 Verma et al. https://creativecommons.org/licenses/by/4.0/ This content is distributed under the terms of the Creative Commons Attribution 4.0 International license.

### Enhanced macrophage activation does not compensate for microglial protective function at early times after infection.

**FIG 6:**
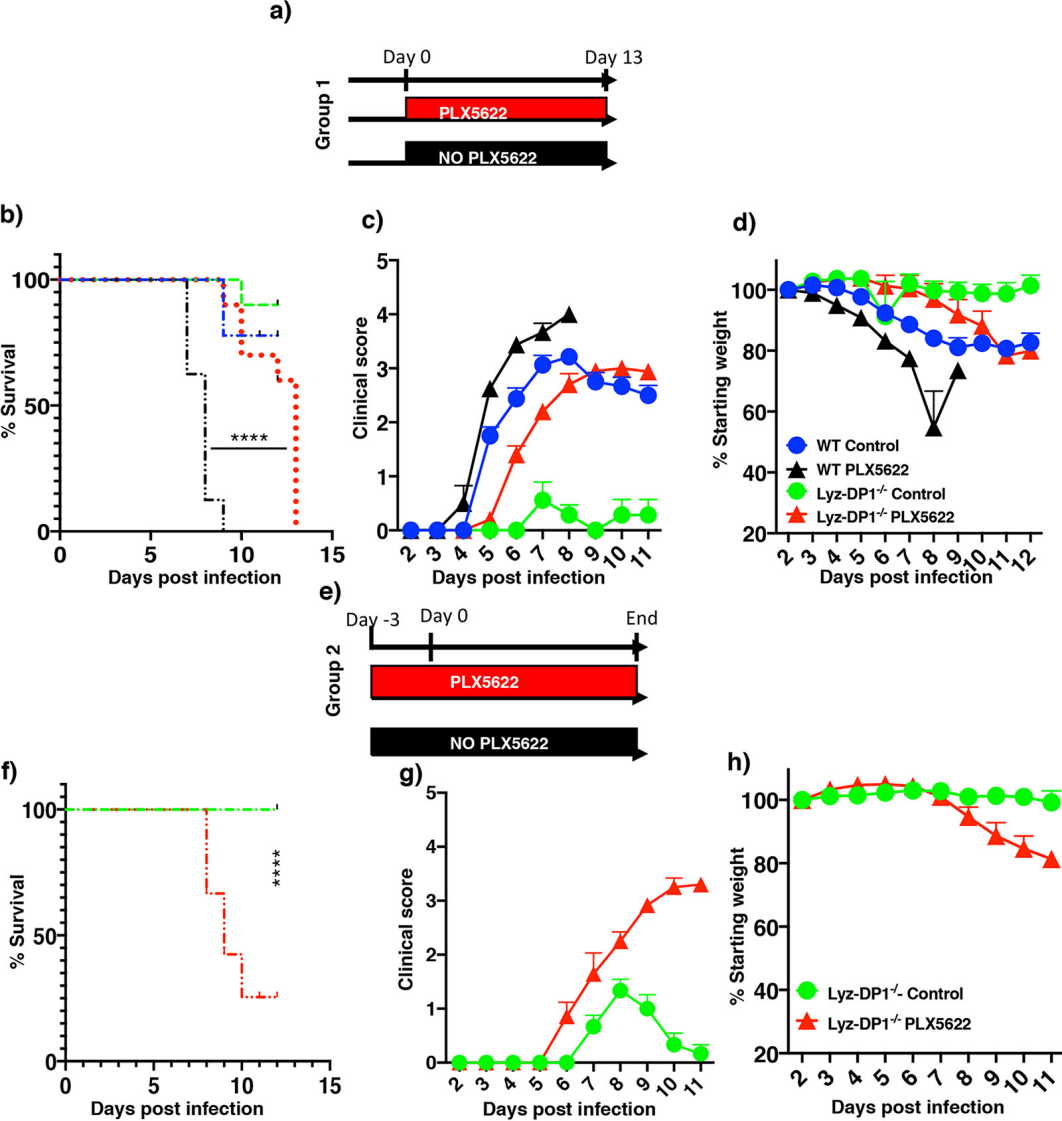
Enhanced macrophage activation modestly compensates for the requirement for microglia in Lyz2-DP1^−/−^ mice. Mice were fed PLX5622-containing or control chow either at the time of infection (group 1) or 3 days prior to infection (group 2). (a to d) Group 1 mice were fed PLX5622 chow on the day of rJHMV infection. (b) A log rank Mantel-Cox test was used to analyze survival differences between PLX5622-treated WT and Lyz2-DP1^−/−^ mice. (c and d) Clinical scores and weights. Data represent pooled results from 2 experiments with 8 to 10 mice per group. A Mann-Whitney U test was used to analyze differences in weight (days 6 to 11) (*P* < 0.05) (d) and clinical score (days 9 to 12) (*P* < 0.05) (c) data. (e to h) Lyz2-DP1^−/−^ mice received PLX5622 chow before infection. Survival (f), clinical scores (g), and weights (h) are shown. A Mann-Whitney U test was used to analyze weight (days 8 to 11) (*P* < 0.05) (h) and clinical score (days 8 to 11) (*P* < 0.05) (g) data. Data are representative of pooled results from 2 experiments with 6 to 7 mice in each group.

We previously showed that microglia depletion prior to or at the time of infection increased mortality after rJHMV infection (6). We next examined whether the augmented macrophage function observed in Lyz2-DP1^−/−^ mice could compensate for the role of microglia at early times postinfection (p.i.). To assess the role of microglia in Lyz2-DP1^−/−^ mice, microglia were depleted by treatment with an inhibitor of CSF1R (colony-stimulating factor 1 receptor) (PLX5622) on the day of infection (group 1) or 3 days prior to infection (group 2), as depicted in Fig. 6a and e. Treatment with PLX5622 results in microglia depletion within 48 h (6). In agreement with previous results, all WT mice treated with PLX5622 at the time of infection died, and all control-treated Lyz2-DP1^−/−^ mice displayed minimal disease. In contrast, PLX5622 treatment of Lyz2-DP1^−/−^ mice resulted in an intermediate phenotype, with increased morbidity compared to control-treated Lyz2-DP1^−/−^ mice (Fig. 6b to d). By day 13 p.i., all PLX5622-treated Lyz2-DP1^−/−^ mice required euthanasia because they reached humane endpoints. Infectious virus was not detected in the brains of these mice. Mice did not recover at these late time points because microglia are required for both initial protection and recovery from infection with rJHMV (29). Prior treatment of all mice with PLX5622 (group 2) resulted in complete lethality. Collectively, these data indicate a critical role for microglia in the initial stages of rJHMV clearance, with only partial compensation by enhanced macrophage function (Fig. 6f to g).

### Macrophage-mediated viral clearance is dependent on microglia.

**FIG 7:**
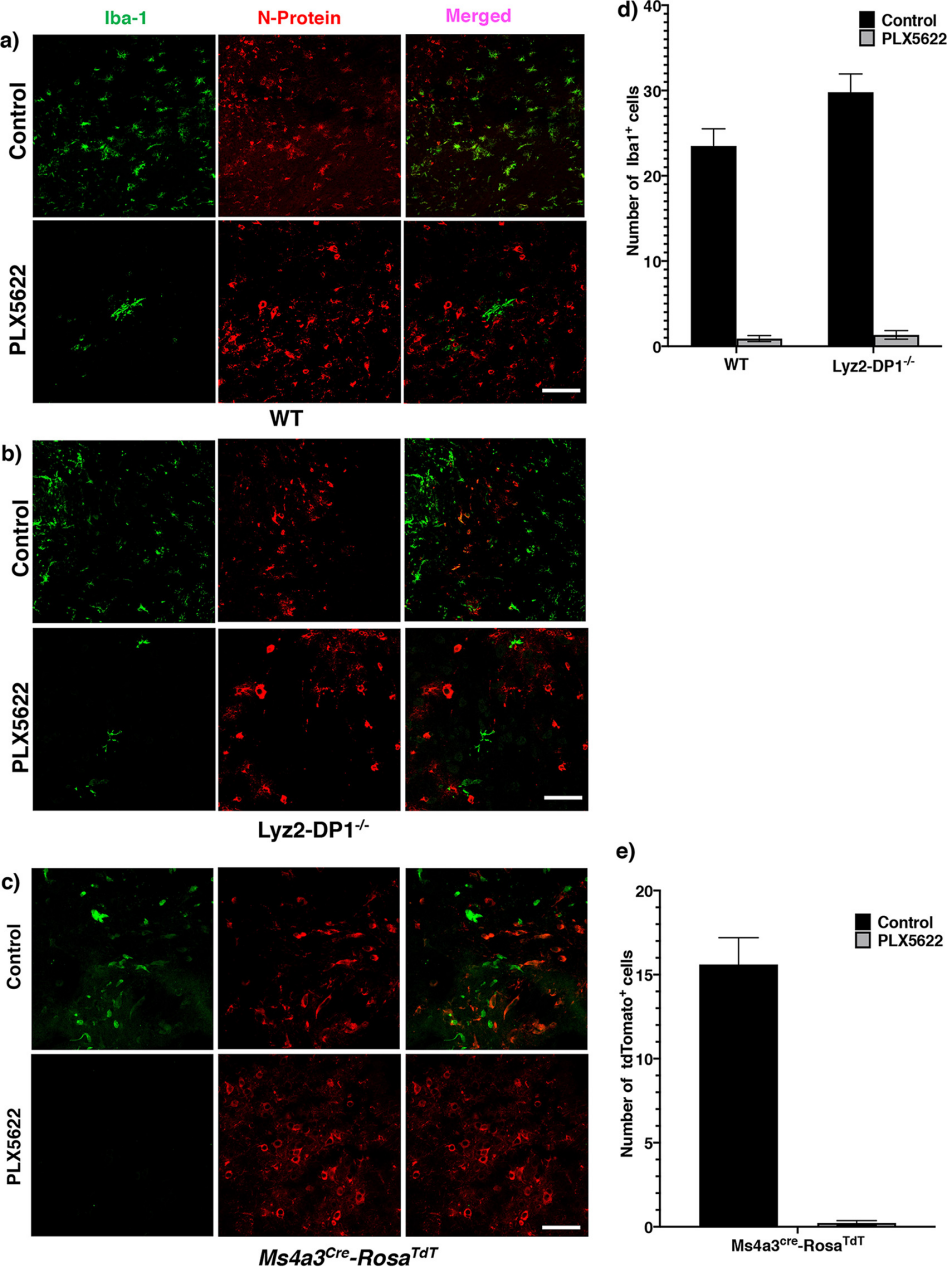
Effects of PLX5622 treatment on macrophage localization in the CNS. Mice were infected in the presence or absence of PLX5622. (a and b) Brains were harvested from WT or Lyz2-DP1^−/−^ mice at 5 dpi and stained for Iba1 (green) and N protein (red). Data are representative of results from three mice in each group. Bars, 50 μm. (c) Ms4a3-cre-tdTomato mice were infected with rJHMV in the presence or absence of PLX5622. Mice were sacrificed at 5 dpi, and brains were harvested and stained for tdTomato (green) to identify macrophages and N protein (red). Data are representative of results from three mice in each group. At least 10 different fields were analyzed per mouse. Bar, 50 μm. (d and e) Summary data. Data were analyzed using Mann-Whitney U tests. Five sections from 3 individual mice were included in the analysis. Data are representative of results for 3 mice per group (means ± SEM).

To begin to understand the role of microglia in facilitating macrophage function, we examined macrophage localization in infected brains. First, we examined Iba1^+^ macrophage/microglia localization in mice fed PLX5622-containing or control chow 3 days prior to infection. While Iba1^+^ cells were located adjacent to infected N^+^ cells in mice fed control chow, there was a nearly complete absence of Iba1^+^ cells in the vicinity of infected cells of mice fed PLX5622 chow (Fig. 7a, b, and d). Notably, PLX5622 treatment did not decrease the total number of CD45^hi^ CD11b^+^ macrophages in the CNS (6). The few remaining cells in the PLX5622-fed mice located near infected cells were morphologically residual microglia and not macrophages. These results suggested that microglia were critical for macrophage localization to the site of infection. Next, to confirm these results, we utilized *Ms4a3-cre* mice, in which, after crossing with B6.Cg-*Gt*(*ROSA*)*26Sor^tm14^*^(^*^CAG-tdTomato^*^)^*^Hze^*/J mice, all granulocyte-monocyte progenitor cells (GMPs) and their lineage, but not microglia, irreversibly express tdTomato (30). At 4 dpi, brains were processed for confocal microscopy and examined for tdTomato-expressing macrophage localization. Consistent with the results obtained using WT mice, virtually no tdTomato^+^ macrophages were detected close to infected cells after PLX5622 treatment (Fig. 7c and e).

## DISCUSSION

Here, we show that PGD_2_/DP1 signaling has strikingly different effects on macrophages and microglia in the brains of mice infected with rJHMV. DP1 signaling in macrophages appears to be anti-inflammatory because in its absence, cells are highly activated and efficacious in virus clearance. LysM is expressed by macrophages and neutrophils (19–21), but our results indicate that the absence of DP1 expression on macrophages was more important for enhanced virus clearance. On the other, in the absence of DP1 signaling in microglia, the immune response is dysregulated, with diminished expression of a type I interferon (IFN-β), an NF-κB-dependent gene (IL-6), and IFN-stimulated genes (ISGs) (MDA5 and RIG-I) and elevated inflammasome activation (IL-1β). Our results also indicate that the phenotype that we observed in mice with microglia-specific DP1 deletion duplicates that observed in mice globally deficient in DP1 signaling, showing that the effects on microglia are dominant, probably because microglia are necessary to initiate the virus-specific immune response. As discussed above, our results are in general agreement with previous studies demonstrating different roles for macrophages and microglia in inflamed brains (17). Macrophages and microglia are derived from different ontogenies, with macrophages being hematogenously derived, while microglia are embryonically derived from the yolk sac and populate the brain early in development (31, 32). Microglia are believed to self-replenish. Whether these differences explain the functional differences that we observe will require additional investigation.

PGD_2_ is the most abundant prostaglandin in the CNS (12), so several studies have focused on its role in neurological disease. Increased levels of DP1 mRNA were detected adjacent to plaques in patients with Alzheimer’s disease compared to normal controls (33). DP1 mRNA was expressed primarily by microglia and astrocytes. Examination of Tg2576 transgenic mice, which develop murine Alzheimer’s disease, showed that DP1 protein was similarly expressed near plaques, consistent with data from human studies (33). In mice with hypoxia-induced encephalitis (HIE), DP1 signaling is deleterious such that endothelial cell degeneration and subsequent outcomes were improved in DP1^−/−^ mice. Furthermore, HPGDS (hematopoietic prostaglandin synthase) (responsible for PGD_2_ production) expression in microglia was increased on autopsy in the brains of neonates who succumbed to HIE compared to those dying from nonneurological etiologies (34). Prominent findings in twitcher mice (lacking GalC expression, expressed by oligodendrocytes) are spasticity, twitching, demyelination, and astrogliosis. Treatment of these mice with an HPGDS inhibitor or crossing of twitcher mice with DP1^−/−^ mice diminished clinical disease and pathological changes (35). In all three cases, DP1 signaling was not anti-inflammatory but rather contributed to the pathogenesis of disease. In contrast, the effects of DP1 signaling are anti-inflammatory in mice with EAE. In the absence of DP1 signaling, less disease occurs. In this instance, deletion of DP1 from dendritic cells (DCs), critical for antigen presentation, resulted in enhanced activation and antigen presentation in lymph nodes. This resulted in T cell apoptosis, fewer cells entering the CNS (36), and milder EAE. Thus, in all of these cases, the absence of DP1 expression resulted in disease amelioration, although only for EAE was the effect shown to result from a reversal of the anti-inflammatory effects of PGD_2_/DP1 signaling, similar to what we observed when DP1 was deleted from macrophages.

After infection of the CNS, microglia are among the first cells to respond, with subsequent infiltration of peripherally derived cells such as NK cells, neutrophils, and macrophages. Although they are phenotypically similar and exhibit similar gene expression profiles, several important functional and genetic differences have been reported when microglia and monocyte-derived macrophages are compared (37–40). Here, to delineate the different roles of PGD_2_/DP1 signaling in these two different cell types, we have used two promoters. CX3CR1, as discussed above, is most abundantly expressed by microglia but also by some macrophages (41). Treatment with tamoxifen will delete DP1 expression by all cell types, but since peripherally derived monocytes and macrophages have a shorter life span than microglia, these newly regenerated monocytes and macrophages will reexpress DP1. Most studies wait at least 4 weeks after tamoxifen treatment to allow the complete replenishment of nonmicroglial cells expressing DP1, but for the reasons described above, this is not feasible in rJHMV-infected mice. Consequently, we infected mice 2 weeks after tamoxifen treatment as a balance between full replacement and maximal susceptibility to infection. We utilized LysM-Cre mice to eliminate DP1 expression by macrophages. LysM-EGFP mice have been utilized previously to differentiate between microglia and macrophages because only a small proportion of microglia expresses enhanced green fluorescent protein (EGFP), and expression levels were reduced compared to those in macrophages (37, 42). LysM may also be expressed by other cell types such as neurons (43). However, DP1 expression in neurons has been reported only in cultured hippocampal neurons (44) and not *in vivo*.

The effects of PGD_2_/DP1 signaling are mediated by cyclic AMP (cAMP). Increased cAMP levels are correlated with diminished phagocytosis and other aspects of macrophage activation (45). Thus, the absence of DP1 on macrophages would be predicted to enhance phagocytosis and macrophage activation, as we observed in rJHMV-infected Lyz2-DP1^−/−^ mice. We observed enhanced expression of TNF and MHC-II antigen as well as phagocytic ability in mice lacking the macrophage-specific expression of DP1. However, our results also show that microglia are required for maximal amelioration of disease, even in the presence of augmented macrophage activation. Consistent with our previous study (6), microglia function is most important at very early times during infection. While microglia depletion prior to infection completely countered the protective effects of macrophage activation observed in Lyz2-DP1^−/−^ mice, depletion at the time of infection had a more nuanced effect on outcomes. Initially, infected Lyz2-DP1^−/−^ mice had slightly milder disease, showing that even in the absence of complete microglia function, the activation of macrophages had an ameliorating effect. However, ultimate outcomes were not improved in these mice because microglia are also required during the recovery stage (29). In their absence, mice remained ill, without evidence of tissue repair. Our data also show that microglia function, at least in part, by recruiting macrophages to sites of infection in the brain. After microglia depletion with PLX5622, virtually no macrophages are located near infected cells. These experiments took advantage of a recently described mouse line, Ms4a3-Cre mice. The Ms4a3 promoter is expressed by granulocyte-macrophage lineage cells but not other hematopoietic cells (30). Few Iba1^+^ cells are detected in the vicinity of infected cells in Lyz2-DP1^−/−^ mice after PLX5622 treatment, consistent with the notion that microglia are required for macrophage recruitment even when macrophages are highly activated. Future work will be directed at determining the molecular basis of microglia-mediated macrophage recruitment to sites of infection.

In conclusion, our results highlight the disparate roles that PGD_2_/DP1 signaling has in macrophages and microglia in the CNS, with implications beyond immune responses to infection. They also show that therapeutic interventions directed at PGD_2_/DP1 signaling need to be designed carefully because these cell-specific effects may confound the interpretation of results.

## MATERIALS AND METHODS

### Mice, virus, and infection.

Six- to seven-week-old C57BL/6N mice were purchased from Charles River Laboratories. DP1^−/−^ mice were previously described (46). Mice with cell-specific DP1 expression were described previously (36). Briefly, PTGDR^flox^ (C57BL/6J background) mice were provided by Richard Breyer, Vanderbilt University. CX3CR1-Cre and Lyz-Cre mice were crossed to PTGDR^flox^ mice to generate CX3CR1 and Lyz conditional PTGDR knockout mice (CX3CR1-PTGDR^−/−^ and LysM-PTGDR^−/−^ mice), respectively. To induce Cre production, 3- to 4-week-old CX3CR1 conditional PTGDR knockout mice and the corresponding control mice were all treated with 3 doses of tamoxifen (10 mg/dose) at 0, 1, and 2 days via oral (p.o.) gavage and then rested for 2 weeks before infection to reduce tamoxifen-caused effects. For infections, mice were lightly anesthetized by administering ketamine/xylazine at 100 μl intraperitoneally (i.p.) and infected intracranially (i.c.) with 700 PFU of the rJHM strain of MHV (47). All animal studies were approved by the University of Iowa Animal Care and Use Committee and met the stipulations of the *Guide for the Care and Use of Laboratory Animals* (48).

### Virus titration.

Mice were sacrificed, and brains were harvested on the indicated days. Tissues were homogenized in phosphate-buffered saline (PBS) using a manual homogenizer and frozen. After thawing, samples were centrifuged, and supernatants were collected. The MHV titer was determined on HeLa-MHVR cells (HeLa cells expressing the MHV receptor) as previously described (49).

### PLX5622 treatment.

PLX5622, provided by Plexxikon Inc., was prepared in an AIN-76A rodent diet at a dose of 1,200 mg/kg standard chow, formulated by Research Diets. Mice were provided PLX5622 in their chow beginning 3 or 0 days before viral infection.

### Isolation of immune cells from brain tissue.

Brains harvested after PBS perfusion were incubated in digestion buffer containing 1 mg/ml collagenase D (Roche) and 0.1 mg/ml DNase I (Roche) at 37°C for 30 min and mechanically dissociated. Brain samples were passed through 70-μm cell strainers, followed by 37% Percoll gradient centrifugation. Mononuclear cells were collected and resuspended in culture medium for further staining.

### Antibodies and flow cytometry.

For flow cytometric staining, we used the following antibodies: CD11b-eFluor 450 (clone M1/70; eBioscience), CD45-phycoerythrin (PE)-Cy7 (clone 30-F11; BioLegend), I-A/I-E-peridinin chlorophyll protein (PerCP)-Cy5.5 (clone M5/114.15.2; BioLegend), TNF-fluorescein isothiocyanate (FITC) (clone MP6-XT22; BD Biosciences), IFN-γ (clone XMG1.2; eBioscience), CD4 (clone RM4-5; BioLegend), and CD8 (clone 53-6.7; eBioscience). For cell sorting experiments, cells were prepared, counted using an automated cell counter, and stained for surface markers. Cells were blocked with 1 μg of Fc block antibody and stained with the indicated antibodies at 4°C. Cells were sorted using a BD FACSAria instrument. For quantification of cytokine-expressing T cells, brain cells were prepared as described above and incubated in Roswell Park Memorial Institute (RPMI) 1640 medium (Invitrogen) with 10% fetal bovine sera (RP10) for 6 h at 37°C with brefeldin A and M133 or S510 peptide to detect virus-specific CD4 or CD8 T cells, respectively. After incubation, cells were fixed, permeabilized using Cytofix/Cytoperm (BD Biosciences), and labeled with CD4, CD8, and anti-IFN-γ. For quantification of TNF-expressing myeloid cells, brain cells were prepared as described above and incubated in RP10 for 6 h at 37°C with brefeldin A. Data were acquired with a BD FACSVerse instrument and analyzed using FlowJo software (TreeStar).

### RNA extraction, PCR, and primers.

RNA was extracted from sorted microglia and macrophages and analyzed by quantitative real-time PCR (qRT-PCR). The primer sets used for PCR were previously described and are listed in Table 1. The expression levels were normalized to hypoxanthine guanine phosphoribosyltransferase (HPRT) by the following threshold cycle (*C_T_*) equation: Δ*C_T_* = *C_T_* of the gene of interest − *C_T_* of HPRT. All results are shown as a ratio to HPRT calculated as 2^−Δ^*^CT^*.

**TABLE 1:** Primers

Target	Direction, sequence
IL-6	Forward, 5′-GAG GAT ACC ACT CCC AAC AGA CC-3′
	Reverse, 5′-AAG TGC ATC ATC GTT CAT ACA-3′

RIG-I	Forward, 5′-CAG ACA GAT CCG AGA CAC TA-3′
	Reverse, 5′-TGC AAG ACC TTT GGC CAG TT-3′

CXCL10	Forward, 5′-GCC GTC ATT TTC TGC CTC AT-3′
	Reverse, 5′-GCT TCC CTA TGG CCC TCA TT-3′

CCL2	Forward, 5′-CTT CTG GGC CTG CTG TTC A-3′
	Reverse, 5′-CCA GCC TAC TCA TTG GGA TCA-3′

IFN-β	Forward, 5′-TCA GAA TGA GTG GTG GTT GC-3′
	Reverse, 5′-GAC CTT TGA AAT GCA GTA GAT TCA-3′

MDA5	Forward, 5′-CGA TCC GAA TGA TTG CA-3′
	Reverse, 5′-AGT TGG TCA TTG CAA CTG CT-3′

HPRT	Forward, 5′-GCG TCG TGA TTA GCG ATG ATG-3′
	Reverse, 5′-CTC GAG CAA GTC TTT CAG TCC-3′

### Confocal imaging.

For immunofluorescence staining, frozen sections were obtained by fixing brains in 4% paraformaldehyde followed by immersion in 10%, 20%, and 30% sucrose solutions for cryoprotection prior to freezing by heat displacement. Sections were processed for citrate-based antigen retrieval (Vector Laboratories) according to the manufacturer’s protocol. Sections were washed three times for 5 min each in PBS before treatment with 0.1% Triton X-100 in PBS for 20 min. The sections were then rinsed in PBS. Next, samples were incubated in CAS-Block (Invitrogen, Thermo Fisher Scientific) followed by incubation with primary antibody diluted in 1% goat serum in PBS overnight at 4°C in a humidity chamber. Primary antibodies against IBA1 (Wako) (1:1,000) and viral N protein (provided by Michael Buchmeier, University of California, Irvine) (1:10,000) were used. Sections were rinsed before incubation with a 1:1,000 dilution of an appropriate Alexa Fluor 546 (A546)- or A488-conjugated goat anti-mouse or anti-rabbit antibody (Thermo Fisher Scientific). After rinsing with PBS, slides were mounted with Vectashield antifade reagent containing 4′,6-diamidino-2-phenylindole (DAPI) (Vector Laboratories). Images were obtained using a Carl Zeiss 710 confocal microscope. Three different areas were imaged for every brain section for cell counting. ImageJ was used for image processing and cell counting.

### *In vivo* antibody treatment for monocyte depletion.

Monocyte migration was blocked by retroorbital injection of anti-CCR2 mAb (clone MC21; 50 μg/dose in 200 μl PBS) administered at −1, 3, and 7 dpi.

### Statistical analysis.

Data were analyzed using a Mann-Whitney U test. A *P* value of <0.05 was considered significant. Statistical significance for survival studies was calculated using the log rank (Mantel-Cox) test with a 95% confidence interval (CI). Results in the graphs are presented as the means ± standard errors of the means (SEM).
