# Supplementary figures and images for: Differential Effects of Prostaglandin D2 Signaling on Macrophages and Microglia in Murine Coronavirus Encephalomyelitis
Source: mBio. 2021 Sep 7;12(5):e01969-21. doi: 10.1128/mBio.01969-21 (PMC8546556; doi:10.1128/mBio.01969-21)

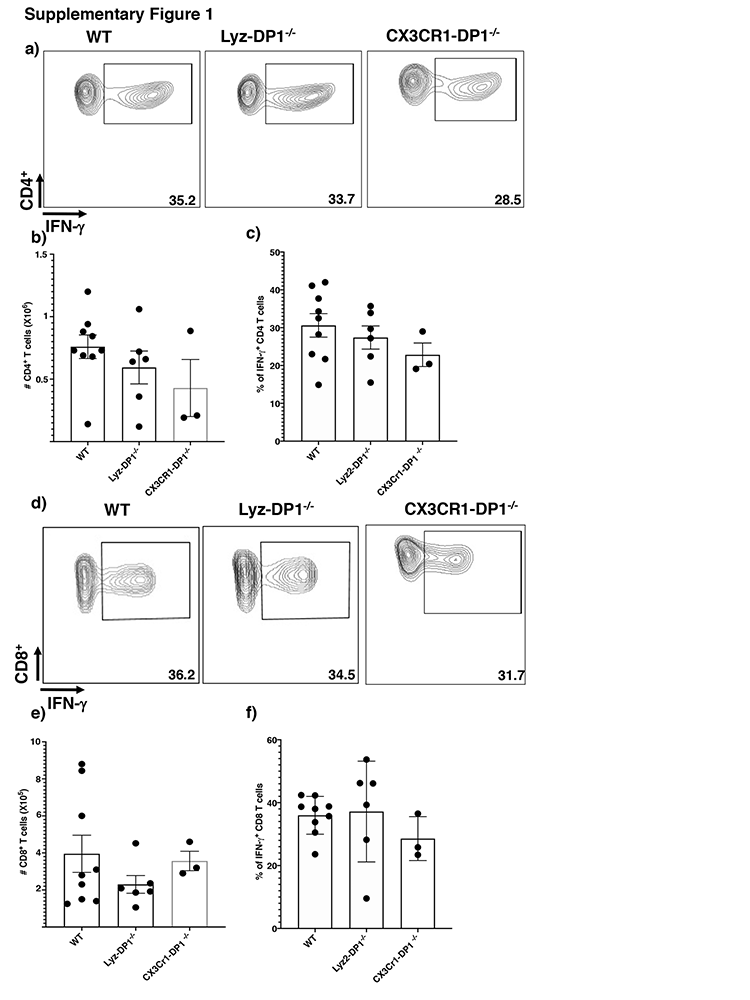

Supplement: FIG S1 [file mbio.01969-21-sf001.tif]

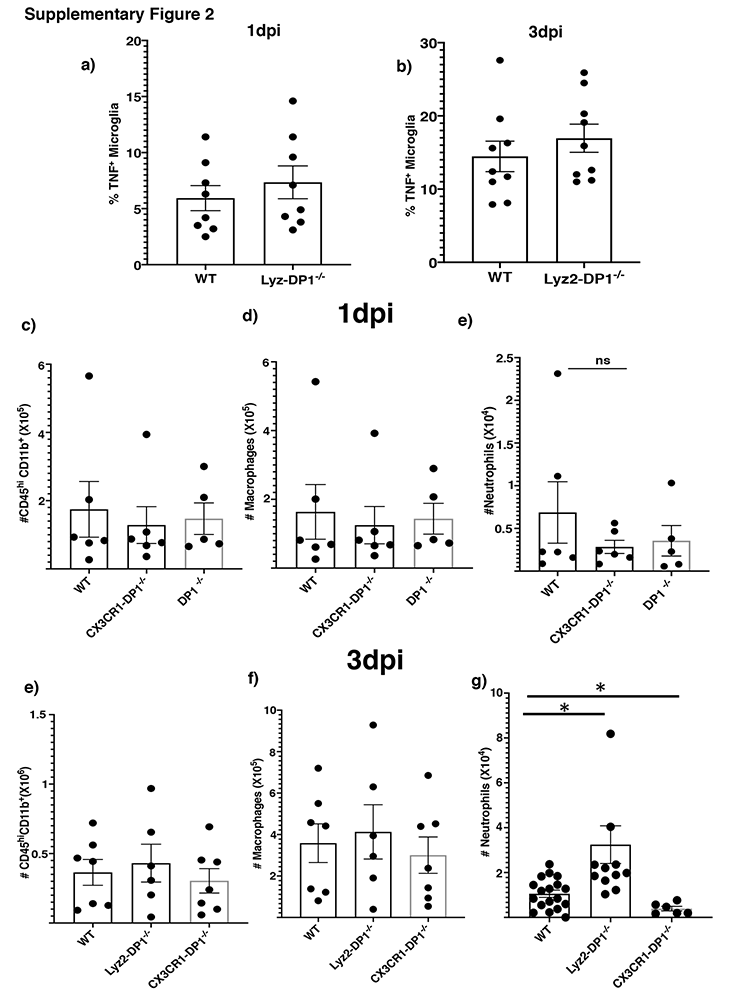

Supplement: FIG S2 [file mbio.01969-21-sf002.tif]

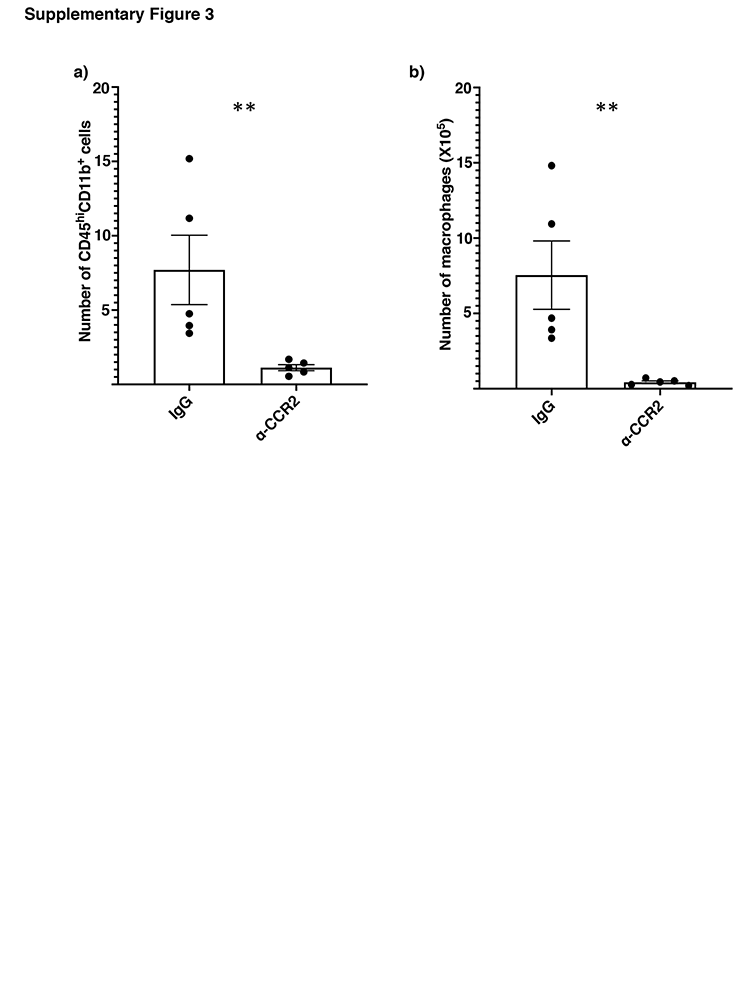

Supplement: FIG S3 [file mbio.01969-21-sf003.tif]
